# Secular trends in mental health profiles among 15-year-olds in Sweden between 2002 and 2018

**DOI:** 10.3389/fpubh.2023.1015509

**Published:** 2023-02-16

**Authors:** Charli Eriksson, Håkan Stattin

**Affiliations:** ^1^Department of Public Health Sciences, Faculty of Social Sciences, Stockholm University, Stockholm, Sweden; ^2^Department of Learning, Informatics, Management and Ethics, Karolinska Institutet (KI), Stockholm, Sweden; ^3^Department of Psychology, Faculty of Social Sciences, Uppsala University, Uppsala, Sweden

**Keywords:** mental health, psychosomatic symptoms, dual-factor model, cluster analysis, secular trends, sex differences

## Abstract

**Background:**

Studies of secular trends in mental unhealth indicate that adolescents in the Nordic countries, especially girls, have an increased reported prevalence of mental health problems the last decades. This increase needs to be seen in the light of the adolescents' assessments of their perceived overall health.

**Objective:**

To investigate whether a person-centered approach to research can enhance understanding of changes over time in the distribution of mental health problems among Swedish adolescents.

**Method:**

A dual-factor approach was used to study changes over time in mental health profiles among nationally representative 15-year-old adolescent samples from Sweden. Cluster analyses of subjective health symptoms (psychological and somatic) and perceived overall health from the Swedish Health Behavior in School-aged Children (HBSC) surveys of 2002, 2006, 2010, 2014, and 2018 were used to identify these mental health profiles (*n* = 9,007).

**Results:**

Four mental health profiles were identified by a cluster analysis which combined all five data collections—Perceived good health, Perceived poor health, High psychosomatic symptoms, and Poor mental health. There were no significant differences in the distributions of these four mental health profiles between the survey years 2002 and 2010, but substantial changes took place between 2010 and 2018. Here, particularly the High psychosomatic symptoms profile increased among both boys and girls. The Perceived good health profile decreased among both boys and girls, and the Perceived poor health profile decreased among girls. The profile involving the most pronounced mental health problems, the Poor mental health profile (perceived poor health, high psychosomatic problems), was stable from 2002 to 2018 among both boys and girls.

**Conclusion:**

The study shows the added value of using person-centered analyses to describe differences in mental health indicators between cohorts of adolescents over longer periods of time. In contrast to the long-term increase in mental health problems seen in many countries, this Swedish study did not find an increase among young persons, both boys and girls, with the poorest mental health, the Poor mental health profile. Rather, the most substantial increase over the survey years, predominantly between 2010 and 2018, was found among the 15-year-olds with High psychosomatic symptoms only.

## Introduction

Adolescents are generally considered healthy, and severe illness and mortality are uncommon. Nevertheless, many young people experience mental health problems in their daily lives during adolescence (1). The greatest burden of disease among young people globally is related to mental health problems (2). Approximately half of the mental health problems that affect people throughout their lifetimes are known to initially manifest themselves by the mid-teenage years (3). Studies of self-reported mental health symptoms among young people have shown a long-term increase over the past 30 years in many countries in northern Europe [for reviews, see (4–6)]. Comparative research has shown that the Nordic countries, especially Norway and Sweden, are among the countries with the largest increases in mental health problems globally (7–11).

In Europe and north America, the prevalence of mental health problems among young people is as high as 35% in representative samples from 2018 (12). Secular trends have been reported and are summarized in several meta-analysis. Rutter and Smith (13) conducted a comprehensive review of the secular trends from the 1950s to the 1990s in the psychosocial disorders of young people. They found evidence of a substantial increase in psychosocial disorders, including depressive disorders, in developed countries. A more recent systematic review of mental health problems in the general adolescent population from 1983 to 2010 (5) concluded that internalizing problems (mental health symptoms) may be increasing, especially among girls, while externalizing problems (such as rule-breaking behavior, drug use and ADHD) appear to be stable. A meta-analysis by Twenge et al. (14) identified a large generational increase in psychopathological symptoms, including depression, among general populations of young people in the US between 1937 and 2007.

A trend of increasing adolescent psychosomatic and depressive symptoms internationally has been reported for non-clinical populations between the 1970s and 2010s. Potrebny et al. (7) found 21 studies with data covering 1982 to 2013 from 36 countries that met the inclusion criteria for their meta-analysis. Their results indicate a weak increasing trend in psychosomatic symptoms in the general adolescent population. The increase was confined to the period from the 1980s to the 2000s and occurred mostly in the northern European region. A recent comparative study of 36 countries suggested that, although psychological and somatic indicators of mental health problems increased slightly between 2002 and 2018, there was no evidence of a global trend, due to great heterogeneity among the countries (15). The increase was mainly found in countries in northern and western Europe. Hence, the temporal trends need to be made more specific. They differed between time periods, countries, and subgroups.

Previous research suggests that the country-specific processes and mechanisms that affect mental health need to be considered (15). This study will use information about mental health indicators from one Nordic country, Sweden. Therefore, previous studies using Swedish data need to be taken into account. In 2010, a systematic review by the Royal Swedish Academy of Sciences noted a lack of Swedish studies of secular trends in adolescent mental health and concluded that it is “not possible to verify or disprove the general perception of a sharply rising frequency of mental disorders among Swedish children and adolescents” (16). A Nordic study including Sweden (NordChild) (9) analyzed psychosomatic symptoms among 7–17-year-olds in four surveys (1984, 1996, 2001, and 2017) and found an increasing trend in symptoms of this kind. Young in Värmland is a survey of Swedish 15–16-year-olds that was conducted eight times between 1988 and 2011 (11, 17). Analyses reveal a trend toward increasing psychosomatic problems, but also different trends for girls and boys.

A more diversified picture of adolescents' health and unhealth is not provided in these studies, because they made use of a bipolar model with one single dimension ranging from lack of symptoms of unhealth to a high prevalence of symptoms. Lack of symptoms of unhealth is not the same as a high level of health (18). Here, the dual-factor model (19, 20) can be used as a guiding principle for ensuring a more complete description of the mental health status of the population.

The dual-factor model of mental health uses two dimensions of mental health simultaneously: one dimension concerns mental illness or psychopathology (subjective symptoms through to psychiatric diseases), while the other dimension concerns wellbeing (subjective wellbeing and health). The model allows for the possibility that an increase in one dimension is not necessarily associated with a decrease in the other. A recent scoping review (20) found empirical support for the dual-factor model; that is, two related factors fitted the data better than one. In the present study, it is assumed that a more complete view of an adolescent's health is obtained by integrating adolescents' reports of psychological and somatic symptoms with their overall perception of health. Such integration is achieved by simultaneously cluster analyzing adolescents' reports of their psychosomatic symptoms and their perceptions of their overall health.

### The current study

The current study applies a person-centered technique using data from Swedish 15-year-olds who participated in five HBSC data collections: 2001/2002, 2005/2006, 2009/2010, 2013/2014, and 2017/2018. In line with the dual-factor model, the current study encompasses two dimensions: one, a non-clinical psychosomatic symptom checklist used in population-based surveys (the HBSC Symptom Checklist, HBSC-SCL), the other a measure of perceived overall health.

Perceived overall health (SRH) is based on an individual's perception and evaluation of her or his overall health. SRH can be distinguished from more specific health constructs in that it captures an overall conception of health, rather than a summation of measures across specific health domains. SRH, as typically operationalized, extends over a continuum ranging from what have been termed “negative” to “positive” health states.

The aim of this study is to explore differences in the distributions of Swedish school-aged adolescents' mental-health profiles or clusters over the years 2002–2018. The intention is to investigate whether analyses of mental health profiles can enhance our understanding of changes in mental health over several years.

## Methods

### Data material

The data were obtained from Swedish Health Behavior in School-aged Children (HBSC) surveys and included 15-year-olds participating in the data collections of 2001/2002, 2005/2006, 2009/2010, 2013/2014, and 2017/2018. The HBSC study comprises cross-sectional data collections of nationally representative samples of adolescents every 4 years. In whichever country it is used, the HSBC follows a standardized protocol for sampling, survey instrumentation and data collection. Data collection is carried out in school classes *via* the self-completion of questionnaires (21). The Swedish Public Health Agency and its predecessors have been responsible for the HBSC in Sweden. The sampling and data collection for the latest surveys were performed by Statistics Sweden. A two-step cluster-sampling design was used for each grade. First, a random, nationally representative sample of schools was drawn, and thereafter, one class in each school that had agreed to participate was randomly selected.

The participation rates and number of participants for the five data collections are given in Table 1. Participation by schools was lower in 2018, but the participation rate among school children in the participating schools was between 81 and 88% during the earlier five data collections. The low school participation level in 2018 was partly due to a restriction laid down by the Swedish Data Protection Agency, which prohibited keeping track of specific schools and reminding them to participate. This restriction was withdrawn for the 2021/2022 HBSC data collection.

**Table 1:** Participation in different years. Percent of participants who were girls or boys are reported within brackets.

	**2002**	**2006**	**2010**	**2014**	**2018**
15-year-olds	1218	1526	2090	2766	1606
Girls	609 (50)	752 (50)	1059 (51)	1358 (49)	777 (48)
Boys	609 (50)	774 (50)	1031 (49)	1408 (51)	829 (52)
School level	84	83	88	77	47

### Measures

**Clustering variables** include two measures. One is the HBSC Symptom Checklist (HBSC-SCL), and the other is perceived overall health.

**The HBSC Symptom Checklist** (HBSC-SCL), also referred to as a measure of ***psychosomatic symptoms***, has been used in all HBSC surveys since 1986. The scale is a non-clinical measure of subjective health symptoms. It poses the stem question, “In the last 6 months, how often have you experienced …?,” followed by eight items: “Headache,” “Stomachache,” “Backache,” “Feeling low,” “Irritability or bad temper,” “Feeling nervous,” “Difficulties in getting to sleep,” and “Feeling dizzy.” The response categories are: (1) “rarely or never,” (2) “about every month,” (3) “about every week,” (4) “more than once a week”, and (5) “about every day.” The symptoms measure has been shown to have acceptable test-retest reliability and internal consistency (22). A recent study of HBSC-SCL using item response theory and differential test function analysis concluded that it was a consistent and one-dimensional scale in two-thirds of the countries where it was used, including the Nordic countries (23).

***Perceived overall health*** was measured by the single item “Would you say your health is …?” Participants were asked to rate their overall health by choosing one of the response categories: (1) “poor,” (2) “fair,” (3) “good,” and (4) “excellent. The question has remained unchanged since the 2001/2002 survey. The time trends in perceived overall health among adolescents in the five Nordic countries have been found to differ (24). Both psychosomatic symptoms and perceived overall health were presented in the reverse order in the questionnaire.

***Sex*** was coded as boy (0) or girl (1).

### Analytic methods

The identification of subgroups from two grouping variables can be either predetermined by cut-offs from median splits or data-driven. We adopted the second approach and performed cluster analysis to identify the naturally occurring patterns/profiles of psychosomatic symptoms and perceived overall health in the samples. Cluster analysis creates groups of people with patterns that are similar to each other and are independent of the median splits of the grouping variables.

A factor analysis of the eight items in the HBSC/SCL produced one factor each survey year, which was then used in further cluster analyses together with the single item on global health. Both measures were standardized. We then applied a hierarchical cluster analysis (Ward's method) to identify the number of clusters. The lower explanatory limit was set at 67% of the total error sums of squares for the number of clusters selected (25). As recommended by Kinder et al. (26), with knowledge of the number of clusters, a non-hierarchical cluster analyses, *K*-means clustering, was used to arrive at the final cluster solution.

First, we combined the data sets and performed one cluster analysis with the same centroid for all years. A cluster analysis for all years combined requires an equal number of persons from each year. The numbers of participants with complete data on the two mental health measures for each of the years from 2002 to 2018 were 1,196, 1,503, 2,030, 2,067, 2,667, and 1,611. Hence, we included all 1,196 persons from year 2002 and randomly selected 1,196 participants from each of the other four survey years. Thereby, the common cluster analysis was based on a total of 5,980 persons. For all cross-tabulations we used the EXACON program, which tests whether a specific cell frequency in a contingency table is larger or smaller than could be expected according to an independence model [the hypergeometric distribution (27)]. A Bonferroni adjusted *p*-value of 0.05 was used to determine which specific cells in the contingency table occurred more often (a Type) and less often (an Antitype) than expected by chance contingency tables (27). The analyses also cover differences between boys and girls.

## Results

### Trends in psychosomatic symptoms and perceived overall health over the study years

We start by reporting the levels of psychosomatic symptoms and perceived overall health for each of the survey years. As seen in Table 2, in all years, girls scored significantly higher than boys on psychosomatic symptoms and lower than boys on perceived overall health (*p* < 0.001). The effect sizes (Cohen's *d*) were medium for psychosomatic symptoms, ranging between −0.61 and −0.69, but were small for overall health, between 0.30 and 0.49. Considering changes over the survey years, psychosomatic symptoms significantly increased from survey year 2002 to survey year 2018 for both boys and girls, but the effect sizes, Cohen's *d*, were small (−0.24 and −0.30). Perceived overall health did not change from survey year 2000 to 2018 for boys but increased somewhat for girls (Cohen's *d* = −0.25). It is in light of these seemingly contradictory trends over the years for psychosomatic and perceived overall health that we adopted the cluster approach in order to identify characteristic profiles that cover both psychosomatic symptoms and perceived overall health.

**Table 2:** Changes over the years 2002 to 2018 in self-rated psychosomatic symptoms and overall health.

	**Psychosomatic symptoms**			**Perceived overall health**		
	**Total**	**Boys**	**Girls**	**Total**	**Boys**	**Girls**
2002	2.27^a^	2.02^ab^	2.50^a^	3.15^a^	3.33^ab^	2.98^a^
2006	2.33^b^	2.07^ab^	2.58^a^	3.19^a^	3.33^ab^	3.06^a^
2010	2.25^a^	2.00^a^	2.51^a^	3.21^b^	3.38^b^	3.04^a^
2014	2.40^c^	2.10^b^	2.68^b^	3.16^a^	3.27^a^	3.05^a^
2018	2.50^d^	2.21^c^	2.77^c^	3.24^c^	3.36^ab^	3.14^b^

Psychosomatic symptoms: Total: F = 26.45, p < 0.001, eta^2^ = 0.01; Boys: F = 9.84, p < 0.001, eta^2^ = 0.01; Girls: F = 16.95, p < 0.001, eta^2^ = 0.01.

Perceived overall health: Total: F = 4.99, p < 0.001, eta^2^ = 0.00; Boys: F = 4.18, p = 0.002, eta^2^ = 0.00; Girls: F = 5.04, p < 0.001, eta^2^ = 0.00.

The superscripts

a, b, c, d represent significant differences (p < 0.05) between survey years in SNK *post-hoc* tests.

### A cluster analysis including all survey years

Combining data across the five survey years yielded a common centroid for all data sets, and a *K*-means cluster analysis of psychosomatic symptoms and perceived overall health resulted in the cluster solution reported in Table 3. Almost half of the adolescents belonged to a Perceived good health profile, which was characterized by a low psychosomatic symptom level and quite high value for perceived overall health. At the other end, the Poor mental health profile included 8% of the 15-year-olds. This profile had a high level of psychosomatic symptoms and a low level of perceived overall health. The High psychosomatic symptoms profile contained 36% of the adolescents and had a high level of psychosomatic symptoms (close to the 0.70 cutoff) and an average level of perceived overall health, while the Perceived poor health profile (7% of the sample) showed a low level of perceived overall health and an average level of psychosomatic symptoms. The proportion of the total variance explained by the four clusters was 72.9%.

**Table 3:** Mental health profiles among 15-year-olds obtained by cluster analysis, aggregated over the years 2002–2018.

**Four cluster profiles:**	**Perceived good health**	**Perceived poor health**	**High symptoms**	**Poor mental health**
Psychosomatic symptoms^b^	−0.78	−0.08	0.67	1.78
Overall health^b^	0.56	−1.80	−0.03	−1.67
*N*	2,902	436	2,171	471
%	48.5	7.3	36.3	7.9
Boys %	63.3^t^	6.6	26.2^a^	3.9^a^
Girls %	34.5^a^	8.0	45.8^t^	11.7^t^

The cluster analysis combines all 5 data collections with N = 1,196 each year. Sex differences are examined with the EXACON program.

t = type, cell frequency more often than expected by chance;

a = antitype, cell frequency less often than expected by chance.

b Low value is < -0.70, Average value is between −0.70 and 0.70, High value is >0.70.

Sex differences: Chi^2^ (3 df) = 530.90, p < 0.001; contingency coefficient = 0.29.

There were significant differences between boys and girls for the set of four mental health profiles (Chi^2^ (3 df) = 596.37, *p* < 0.001). An EXACON analysis showed that Perceived good health was more common among boys while High psychosomatic symptoms and Poor mental health were more common among girls. The most obvious sex difference was that, whereas about two of three boys belonged to the Perceived good health profile, this was the case for only a minority of the girls, 35%. In fact, belonging to the High psychosomatic profile was more common among girls than belonging to the Perceived good health profile. There were no sex differences for the Perceived poor health profile, but it was three times more common for girls than boys to belong to the Poor mental health profile (12 vs. 4%).

### Changes in mental health profiles from 2002 to 2018

**Figure 1:**
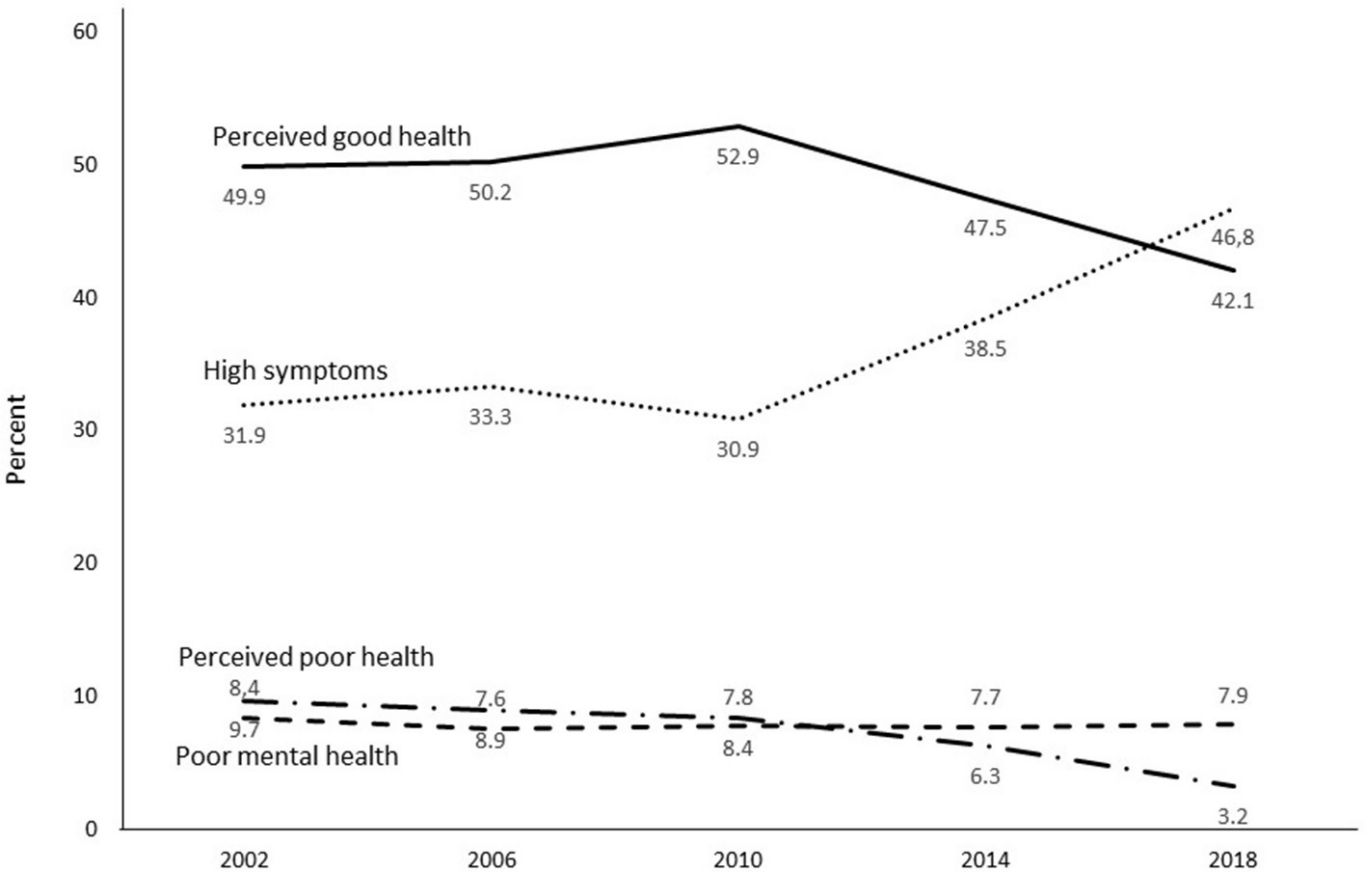
Secular trends from 2002 to 2018 for four mental health profiles among representative samples of 15-year-old adolescents.

As a first step toward identifying stability and change in the four mental health profiles, the distributions of the profiles were plotted over the five survey years. Figure 1 reports these plots for the total sample. They indicate that stability characterized the trends of all four mental health profiles from year 2002 to year 2010, but that more substantial increases and decreases took place between 2010 and 2018. Follow-up analyses testing these changes between 2002 and 2010 and between 2010 and 2018 are reported in Table 4. For the total sample there were no significant changes in the distributions for the mental health profiles between year 2002 and year 2010. By contrast, there were significant decreases for Perceived good health and Perceived bad health profiles, and a significant increase for the High psychosomatic symptoms profile between the year 2010 and year 2018. There was no significant change in the Poor mental health profile between these two survey years. Overall, these results indicate that the window for changes in the mental health profiles was between the survey years 2010 and 2018.

**Table 4:** Comparisons of the distributions of the four mental health profiles between 2002 and 2010 and between 2010 and 2018 for the total samples and for boys and girls separately.

	**Perceived good health**	**Perceived bad health**	**High symptoms**	**Poor mental health**
**Total sample:**				
2002	49.9	9.7	31.9	8.4
2010	52.9	8.4	30.9	7.8
Chi^2^ (3df) = 2.76, *p* = 0.430, Cramér's *V* = 0.03				
2010	52.9^t^	8.4^t^	30.9^a^	7.8
2018	42.1^a^	3.2^a^	46.8^t^	7.9
Chi^2^ (3df) = 81.31, *p* < 0.001, Cramér's *V* = 0.18				
**Boys:**				
2002	64.5	8.3	23.5	3.7
2010	68.3	6.3	21.8	3.6
Chi^2^ (3df) = 2.64, *p* = 0.451, Cramér's *V* = 0.05				
2010	68.3^t^	6.3	21.8^a^	3.6
2018	57.2^a^	3.8	34.9^t^	4.1
Chi^2^ (3df) = 27.27, *p* < 0.001, Cramér's *V* = 0.15				
**Girls:**				
2002	35.6	11.1	40.2	13.1
2010	37.2	10.5	40.3	12.0
Chi^2^ (3df) = 0.59, *p* = 0.899, Cramér's *V* = 0.02				
2010	37.2^t^	10.5^t^	40.3^a^	12.0
2018	29.1^a^	2.7^a^	57.0^t^	11.3
Chi^2^ (3df) = 51.20, *p* < 0.001, Cramér's *V* = 0.21				

The table shows the percentages for each of the four mental health profiles for respective survey year.

t = type, cell frequency higher than expected by chance;

a = antitype, cell frequency lower than expected by chance.

**Figure 2:**
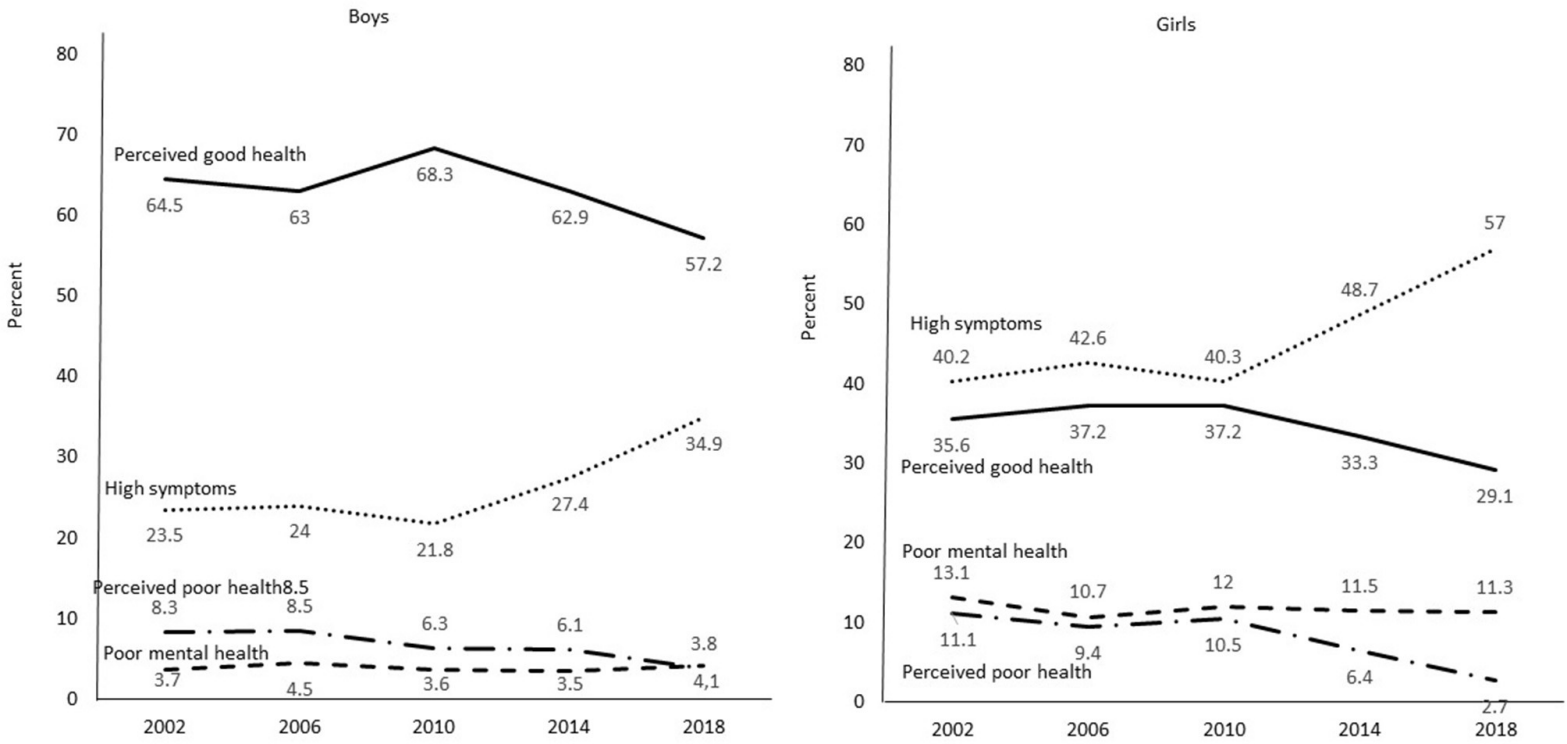
Secular trends from 2002 to 2018 for four mental health profiles among representative samples of 15-year-old boys and girls.

This also seems to be the case when analyzing the secular trends of the mental health profiles separately for boys and girls. As shown by the plots in Figure 2, the distributions of the four mental health profiles did not change much between survey years 2002 and 2010 for either boys or girls. As reported in Table 4, for both boys and girls, no significant changes in the distributions of the four mental health profiles were found between 2002 and 2010. However, there were significant differences between the survey years 2010 and 2018. Significant decreases were found for the Perceived good health profile and increases for the High psychosomatic symptoms profile for both sexes. A decrease in the Perceived poor health profile was found for girls. These findings suggest, first, that when significant changes over time occur in a mental health profile for one sex, they also occur for the other sex (with the exception of no significant differences for the Perceived poor health profile among boys). Second, the changes in distributions of the mental health profiles took place primarily between 2010 and 2018 for both sexes. Of note is that there were no significant changes in the distributions of the Poor mental health profile either from 2002 to 2010 or from 2010 to 2018.

As seen in Figure 2, already in 2010 it was considerably more common for boys to belong to the Perceived good health profile than for girls (68 vs. 37%), and more common for girls to belong to the High psychosomatic symptoms profile than for boys (40 vs. 22%). Girls were also more likely to belong to the Poor mental health profile (12 vs. 4%). The changes that took place between 2010 and 2018 seems to occur to the same extent for both girls and girls. The decrease in Perceived good health over this time span (from 68 to 57% for boys, and from 37 to 29% for girls) amounts to 16% for girls and 22% for boys. The strong increase in High psychosomatic symptoms (from 22 to 35% among boys and from 40 to 57% among girls) was actually higher for boys (60%) than for girls (41%). Also, there was a small increase in the Poor mental health profile for boys (14%) but a small decrease for girls (6%). However, the decrease in Perceived poor health was considerably larger for girls (11 to 3%), which is an increase of 74%, than for boys (6 to 4%), which is a decrease of 40%. In sum, there were major sex differences for the mental health profiles, Perceived good health, High psychosomatic symptoms and Poor mental health, already in the survey year 2010. The changes that took place between 2010 and 2018 were generally about the same for girls and boys, with the result that the sex differences in 2018 were similar to those that prevailed in 2010.

### Mental health profiles for each survey year

When the data sets from the five survey years were analyzed separately, the cluster analyses resulted in four mental health profiles for each of these years (see Supplementary Table S1). Three of them—Perceived good health, High psychosomatic symptoms, and Poor mental health—were present for all years. The profile Perceived poor health was present for the first four survey years. However, the cluster analysis of the 2018 sample generated an Average health profile instead, with average levels of both psychosomatic symptoms and perceived overall health. In short, the cluster analyses for each of the five survey years were, with one exception, similar to the cluster analysis that combined all 5 years.

## Discussion

Previous studies have shown increased mental health problems among adolescents, particularly among girls (4–7). The increase has been reported on in studies using adolescent as well as parental reports (9). International (1, 8, 15), Swedish HBSC studies (16, 28), and regional Swedish studies (11, 17) have all reported more problematic mental health over the years among adolescents. The present study shows an increase in psychosomatic symptoms from 2002 to 2018, but also a slight increase in perceived overall health over the same years. How this translates into normally occurring patterns of psychosomatic symptoms and perceived overall health was examined here.

This study used cluster analysis to shed new light on what has happened to adolescents' perceptions of their mental health in Sweden between 2002 and 2018. We simultaneously cluster-analyzed 15-year-old adolescents' reports on their psychosomatic symptoms and perceived overall health in the years 2002, 2006, 2010, 2014, and 2018 (*n* = 5,980). Four clusters were identified when combining all five data sets: Perceived good health (quite high level of perceived overall health and low level of psychosomatic symptoms), Perceived poor health (low level of perceived overall health and average level of psychosomatic symptoms), High psychosomatic symptoms (high level of psychosomatic symptoms and average level of perceived overall health), and Poor mental health (low level of overall health and high level of psychosomatic symptoms). With one exception, cluster analyses performed for each of the five survey years also produced the same four types of mental health profiles. These latter cluster analyses show that the four mental health profiles in the study remained intact over many years, from 2002 to 2018.

Few differences in the distributions of these four mental health profiles were found between the survey years 2002 and 2010, but substantial increases and decreases took place between 2010 and 2018. For the total sample, there was a substantial increase over these years for belonging to the High psychosomatic profile, a substantial decrease for belonging to the Perceived good health profile, and a decrease for belonging to the Perceived poor health profile. In sum, the changes that took place in the cluster profiles over the study years occurred chiefly from 2010 to 2018.

In contrast to the common findings of increased mental health problems over time, particularly among girls (15, 17, 28), we did not find any changes over the years 2002 to 2018 in the proportion of adolescents in the cluster characterized by the poorest mental health—having both High psychosomatic symptoms and low perceived overall health. This means that the proportion of adolescents with the most serious form of mental ill-health appears not to have changed much over the years covered by the study.

The different developmental trends for Poor mental health and High psychosomatic symptoms should be noted. The Poor mental health profile did not change much in size over the years. It included about 12% girls and 4% boys. In a non-clinical sample of 15-year-olds such as ours, it was expected that the serious mental ill-health group would be small (2, 7). By contrast, the High psychosomatic symptoms profile, with young people having High psychosomatic symptoms but an otherwise average level of perceived overall health, increased between 2010 and 2018 among both boys and girls. Here, it appears necessary to both differentiate between and combine information about the two health indicators. First, there was only a modest negative correlation of −0.40, *p* < 0.001 between self-rated psychosomatic symptoms and perceived overall health. Thus, they do not measure opposite things. Second, of all the 15-year-olds in the clusters that were characterized by High psychosomatic symptoms—the Poor mental health cluster and the High psychosomatic symptoms cluster—there were less than one in five who combined high levels of psychosomatic symptoms with low perceived overall health. Potentially, the Poor mental health profile encompasses clinical conditions that can account for an important part of the burden of disease among young people (2). A recent validation of the four mental health profiles for Swedish 15-year-olds in 2018, comparing measures of a positive self (mental wellbeing, self-esteem, and general self-efficacy), positive school experiences, and perceived social support from parents and friends, found the adolescents in the poor mental health profile to have considerably lower levels on these measures than the adolescents in the three other mental health profiles (23).

Note that of the two clusters in the current study, High psychosomatic symptoms and Poor mental health, the latter appears as the cluster of adolescents in particular need of attention and support from school health services and other treatment facilities. Further analyses of these adolescents regarding possible chronic conditions, psychiatric disorders, and pharmacologic and psychotherapeutic treatments would be beneficial. One hypothesis is that the adolescents with High psychosomatic symptoms and average perceived overall health still have the coping skills needed to navigate their everyday life environments, but that this might change if their perceived overall health is affected.

We can only speculate about what contributed to the increase in the mental profile characterized by High psychosomatic symptoms and the decrease in perceived good health among the adolescents that took place between 2010 and 2018. One characteristic of this time period was the strong emergence of adolescents' encounters with social media. In Sweden 2010 the majority of adolescents used electronic media communication (EMC) 5 days or more (29). Social media use impacts social and emotional wellbeing in a negative way when it has addiction-like symptoms (30, 31). Social media also has had the result that bullying at school extends to cyber bullying. Further research on the relationship between EMC and mental health might be facilitated by person-centered analyses with regard to both EMC and mental health.

### Sex differences

There was little evidence that problematic mental health increased more for girls than for boys over the survey years. Sex differences in the indicator of mental unhealth among adolescents in the present study—psychosomatic symptoms—need to be seen in light of what happened between survey years 2002 and 2018. Already in 2002, girls scored higher on psychosomatic symptoms than did boys. Cohen's *d* was −0.64. In 2018, again girls scored higher for psychosomatic symptoms than boys, and Cohen's *d* was about the same, −0.69. What happened between the years 2002 and 2018 was that the level of psychosomatic symptoms increased to about the same extent for both boys and girls, and the sex differences that appeared in 2002 reappeared at about the same level 16 years later.

A similar tendency over the years pertains to the mental health profile, High psychosomatic symptoms. Most of the changes in this profile took place between 2010 and 2018. More girls than boys belonged to the High psychosomatic symptoms profile in 2010 (40 vs. 22%). There was a substantial increase in being a member of this profile from 2010 to 2018 for both boys and girls (an increase of 60 and 41%, respectively). Again, the same strong sex difference also existed in 2018: 57% of girls and 35% of boys then belonged to this mental health profile. Concerning the Poor mental health profile, there were few changes over the whole period from 2002 to 2018. In 2002, 4% of boys and 12% of girls belonged to this profile, while 16 years later 4% of the boys and 11% of the girls belonged. In sum, there were substantial sex differences for psychosomatic symptoms and the two mental health profiles with high levels of psychosomatic symptoms in 2002. Sixteen years later, the magnitudes of these differences between boys and girls were about the same. The changes that took place in psychosomatic symptoms and the two mental health profiles with high levels of psychosomatic symptoms between 2002 to 2018 were of the same magnitude for boys and girls (or lack of change over time for the Poor mental health profile).

The opposite is true for the Perceived good health profile. Considerably more boys than girls belonged to this profile in 2010: a majority of boys, 68%, but only a minority of girls, 37%. There were decreases in belonging to this profile among both boys and girls (a decrease of 16 and 22%, respectively). In the end, in 2018, about the same sex difference prevailed as in 2010 (57% of boys and 29% of the girls belonged to this profile). The low figure for the Perceived good health for girls is noteworthy. In fact, more girls belonged to the High psychosomatic symptoms profile than to the Perceived good health profile during all the years. All in all, the sex differences that existed for the Perceived good health, High psychosomatic symptoms, and Poor mental health prevailed over the years. When increases or decreases in the distributions of the mental health profiles changed for one of the sexes, they also changed for the other sex to about the same extent over the same time.

One further observation, that we have little explanation for, is that the proportion of girls who belonged to the Perceived poor health profile decreased substantially from 2010 to 2018 (from 11 to 3%), and more than for boys (from 6 to 4%), at a time when, simultaneously, the High psychosomatic symptoms profile increased, and the Perceived good health profile decreased substantially among girls.

The sharp focus on the increase in mental health problems among girls over the last decades might have had the consequence that the link between girls' and boys' reports of mental health problems has gone unnoticed. Undoubtedly, girls report considerable higher levels of mental health problems than boys, but the points in time for increases and decreases in mental health profiles were found to be very similar for the sexes in this study, and the rates of these changes over time were also similar. Theoretically, this might mean that the evocative conditions for changes in the distribution of mental health profiles over time can be quite similar for girls and boys. The question then is not what has contributed to changes in girls' mental health problems, but what contributed to the changes in both girls' and boys' mental health problems. The answer might indicate that the evocative conditions behind the secular trends for mental health problems might not be unique to girls but could cover conditions that affect both sexes. These are speculations, but they offer another entry point into the interpretation of the secular trends in mental health problems among adolescents that have been seen over longer periods of time.

### Strengths and weaknesses

The present cluster analysis of Swedish 15-year-olds' mental health problems over time provides better understanding of the windows in time when changes in these problems have occurred, and not occurred, and gives more information on which profiles of mental health problems have increased and which have remained stable or decreased.

A major strength of the study is that the same study protocol was used for the variables included in the present analyses at all data collections. The measures used have good validity and reliability according to previous research (12, 15, 21). The participation rate at individual level was consistently high, although the participation rate of schools decreased in 2017/2018. The identified profiles were similar across the five data collections and the results are statistically robust. The monitoring of mental health among adolescents is based on self-reports, which may be a weakness, but it is essential for understanding adolescent mental health through the eyes of adolescents themselves, in line with the UN Child Convention. Further interesting research would explore which protective or risk factors are important for being in not just the Perceived good health cluster or the Poor mental health cluster but also the High psychosomatic symptoms and Perceived poor health clusters.

A weakness of the study is that it only investigates Swedish 15-year-olds. As decreased mental health among adolescents has been observed in other northern European countries (4–6, 8–11), it would be of interest to extend the analysis to more countries; a further cluster analysis for five Nordic countries combined is planned.

The cross-sectional design is a further limitation of the study; only longitudinal studies enable the analysis of causal inferences. However, when analyzing trends, measuring mental health for representative samples of adolescents over time in different countries can effectively trace secular trends. The study used non-clinical measures of subjective health. Future studies need to compare the findings with screening and diagnostic instruments used in clinical settings.

## Conclusion

The present study used person-centered analysis to describe inter individual variations in adolescent mental health over repeated cross-sectional samples of 15-year-olds from 2002 to 2018. Cluster analysis found four distinct mental health profiles, based on levels of psychosomatic symptoms and perceived overall health, in all but one sample: Perceived good health, Perceived poor health, High psychosomatic symptoms, and Poor mental health. The last group of adolescents, with the most serious form of ill-health, both high levels of psychosomatic symptoms and low levels of overall health, made up about 8% of the sample at each data collection from 2002 to 2018 (around 4% of the boys and 12% of the girls). For the other three mental health profiles, changes took place primarily between the years 2010 and 2018. The most notable increases over these years were seen among girls and boys who had a High psychosomatic symptom load but were otherwise still content, i.e., the High psychosomatic symptoms group, from 22 to 35% among boys, and from 40 to 57% among girls. The Perceived good health group decreased among boys and girls over these years (from 68 to 57% of the boys, and from 37 to 29% of the girls). Also, the Perceived poor health group, with low levels of perceived overall health but average levels of psychosomatic symptoms, decreased among both boys (6 to 4%) and girls (11 to 3%) over the later years. Apparently, the increase in mental health problems between 2002 and 2018 among Swedish 15-year-olds took place between 2010 and 2018 primarily among adolescents who displayed high levels of psychosomatic symptoms but otherwise had average levels of perceived overall health. There were no changes over the years 2002 to 2018 in the proportion of adolescents who belonged to the cluster Poor mental health. Already 2002 three times as many girls belonged to this cluster than boys.

## Data availability statement

Open access to the data on the mandatory questions in the 2018 HBSC survey cycle can since October 2022 be achieved through the HBSC Open Access portal. More information can be found on the webpage: https://hbsc.org/data/.

## Ethics statement

Ethical review and approval was not required for the study on human participants in accordance with the local legislation and institutional requirements. Written informed consent for participation was not provided by the participants' legal guardians/next of kin because the study was conducted according to the Guidelines of the Declaration of Helsinki. The Swedish study using HBSC data is deemed exempt from human subject research review by the Regional Ethical Review Board in Stockholm.

## Author contributions

CE and HS designed the study. CE drafted the manuscript. HS performed the analyses. Both authors have reviewed, edited the manuscript, read, and agreed to the published version of the manuscript.
