# Supplementary material for: Secular trends in mental health profiles among 15-year-olds in Sweden between 2002 and 2018
Source: Front Public Health. 2023 Feb 16;11:1015509. doi: 10.3389/fpubh.2023.1015509 (PMC9978413; doi:10.3389/fpubh.2023.1015509)
Supplement: Supplementary file 1 [file Table_1.DOCX]

Table S1: Mental health profiles among 15-year-olds from 2002 to 2018. Separately for each year.

| 2002 |  | Perceived good health | Perceived poor health | High psychosomatic symptoms | Poor mental health |
| --- | --- | --- | --- | --- | --- |
|  | Symptoms | -0.81 | -0.14 | 0.66 | 1.58 |
|  | Perceived health | 0.62 | -1.63 | 0.02 | -1.70 |
|  | N | 561 | 97 | 419 | 119 |
|  | % | 46.9 | 8.1 | 35.1 | 10.0 |
|  | Boys % | 61.8 | 7.4 | 26.2 | 4.6 |
|  | Girls % | 32.3 | 8.8 | 43.7 | 15.2 |
| 2006 |  | Perceived good health | Perceived poor health | High psychosomatic symptoms | Poor mental health |
|  | Symptoms | -0.74 | -0.22 | 0.74 | 1.75 |
|  | Perceived health | 0.56 | -1.70 | 0.01 | -1.71 |
|  | N | 754 | 131 | 496 | 122 |
|  | % | 50.2 | 8.7 | 33.0 | 8.2 |
|  | Boys % | 65.3 | 8.8 | 21.5 | 4.3 |
|  | Girls % | 38.8 | 8.5 | 41.6 | 11.1 |
| 2010 |  | Perceived good health | Perceived poor health | High psychosomatic symptoms | Poor mental health |
|  | Symptoms | -0.73 | -0.12 | 0.66 | 1.80 |
|  | Perceived health | 0.57 | -1.76 | -0.03 | -1.71 |
|  | N | 1021 | 157 | 680 | 174 |
|  | % | 50.5 | 7.3 | 31.0 | 8.6 |
|  | Boys % | 66.2 | 6.6 | 23.5 | 3.7 |
|  | Girls % | 34.2 | 8.9 | 43.5 | 13.4 |
| 2014 |  | Perceived good health | Perceived poor health | High psychosomatic symptoms | Poor mental health |
|  | Symptoms | -0.77 | -0.08 | 0.71 | 1.82 |
|  | Perceived health | 0.52 | -1.88 | -0.05 | -1.84 |
|  | N | 1315 | 171 | 1014 | 169 |
|  | % | 49.3 | 6.4 | 38.0 | 6.3 |
|  | Boys % | 65.6 | 6.5 | 24.9 | 3.0 |
|  | Girls % | 33.9 | 6.3 | 50.3 | 9.5 |
| 2018 |  | Perceived average health | Perceived poor health | High psychosomatic symptoms | Poor mental health |
|  | Symptoms | -0.56 | -0.60 | 1.02 | 1.11 |
|  | Perceived health | -0.43 | 1.19 | -0.22 | -2.15 |
|  | N | 532 | 502 | 449 | 129 |
|  | % | 33.0 | 31.1 | 27.9 | 8.0 |
|  | Boys % | 39.1 | 40.8 | 15.3 | 4.8 |
|  | Girls % | 27.6 | 22.5 | 39.1 | 10.9 |

*Note.*

Guidance for interpretation: Low value is < -0.70, Average value is between -0.70 and 0.70, High value is > 0.70.

The percent of the total variance explained for 4 clusters were: 2002 (72.8%), 2006 (71.7%), 2010 (73.4%), 2014 (69.8%), and 2018 (72.4%).

Gender differences: Chi^2^ (3 df): 2002: 118.13, *p* < .001; 2006: 120.50, *p* <.001; 2010: 223.38, *p* < .001; 2014: 292.08, *p* < .001; 2018: 155.10, *p* < .001.
